# P46 - Neck circumference may be related with severe asthma in children

**DOI:** 10.1186/2045-7022-4-S1-P101

**Published:** 2014-02-28

**Authors:** Süleyman Tolga Yavuz, Bülent Hacıhamdioğlu, Mutluay Arslan

**Affiliations:** 1 Department of Pediatric Allergy, Gulhane Military School of Medicine, Ankara, Turkey; 2 Department of Pediatric Endocrinology, Gulhane Military School of Medicine, Ankara, Turkey; 3 Department of Pediatrics, Gulhane Military School of Medicine, Ankara, Turkey

## Background

Obesity is an established risk factor for asthma in children. Measures of central obesity are reported to be more associated with the severity of asthma in adults. The aim of the study is to investigate the association between fat distributions which is determined by anthropometric measures including neck circumference and asthma severity in children.

## Method

Children with asthma who were followed in our outpatient department of pediatric allergy unit were consecutively recruited. Asthma severity was graded according to NAEPP guidelines. Patients were categorized into two groups; children with intermittent and mild persistent asthma formed Group 1 (mild asthma) whereas children with moderate and severe persistent asthma formed Group 2 (severe asthma). Anthropometric measures including height, weight, neck circumference (NC), waist circumference and hip circumference were obtained. Pulmonary function tests and skin prick tests were performed in all subjects.

## Results

A total of 127 children (82 male, 64.6%) with a median age of 8.3 (6.4-11.3) years were included. Aeroallergen sensitization was present in 77 (60.6%) patients. 91 patients (71.6) were in the mild asthma group. There were no significant difference between two groups in terms of age, gender, aeroallergen sensitization, obesity prevalence, body mass index, waist and hip circumferences. NC of children with severe asthma were significantly wider than children with mild asthma (29.0 cm (27.0-32.0) vs. 28.0 (26.0-30.0), p=0.019). The prevalence of children with NC higher than 90th percentile were also more frequent in children with severe asthma (15 (41.7%) vs. 21 (23.1%)). Result of multivariate logistic regression analysis revealed that presence of NC > 90th percentile were associated with severe asthma in children (odds ratio; [95% confidence interval] (2.63 [1.10–6.28]; p=0.029).

## Conclusion

Neck circumference, which is a simple tool of anthropometric measures, is more associated with asthma severity in children when compared with standard methods.

